# Associations between Subjective Happiness and Dry Eye Disease: A New Perspective from the Osaka Study

**DOI:** 10.1371/journal.pone.0123299

**Published:** 2015-04-01

**Authors:** Motoko Kawashima, Miki Uchino, Norihiko Yokoi, Yuichi Uchino, Murat Dogru, Aoi Komuro, Yukiko Sonomura, Hiroaki Kato, Shigeru Kinoshita, Masaru Mimura, Kazuo Tsubota

**Affiliations:** 1 Department of Ophthalmology, Keio University School of Medicine, Tokyo, Japan; 2 Department of Ophthalmology, Kyoto Prefectural University of Medicine, Kyoto, Japan; 3 Department of Neuropsychiatry, Keio University School of Medicine, Tokyo, Japan; University of British Columbia, CANADA

## Abstract

**Importance:**

Dry eye disease has become an important health problem. A lack of concordance between self-reported symptoms and the outcome of dry eye examinations has raised questions about dry eye disease.

**Objective:**

To explore the association between subjective happiness and objective and subjective symptoms of dry eye disease.

**Design:**

The study adopted a cross-sectional design.

**Setting:**

All the employees of a company in Osaka, Japan.

**Participants:**

672 Japanese office workers using Visual Display Terminals (age range: 26–64 years).

**Methods:**

The dry eye measurement tools included the Schirmer test, conjunctivocorneal staining, the tear film break-up time, as well as the administration of a dry eye symptoms questionnaire. Happiness was measured by the Subjective Happiness Scale.

**Main Outcome Measures:**

Dry eye examination parameters, dry eye symptoms questionnaires, and the Subjective Happiness Scale score.

**Results:**

Of the 672 workers, 561 (83.5%) completed the questionnaires and examinations. The mean Subjective Happiness Scale score was 4.91 (SD = 1.01). This score was inversely correlated with the dry eye symptom score (r = -0.188, p < 0.001), but was not associated with objective findings which include conjunctivocorneal staining, low Schirmer test score, or low tear film break-up time. The level of subjective happiness was the lowest in the group without objective results, but reported subjective symptoms of dry eyes (p < 0.05).

**Conclusions and Relevance:**

There is evidence of the relationship between subjective happiness and self-reported symptoms of dry eyes. Findings of this study revealed a new perspective on dry eye disease, including the potential for innovative treatments of a specific population with dry eye disease.

## Introduction

The high and increasing prevalence of dry eye disease (DED) has become an important public health problem, especially in developed countries with advanced information technology and aging populations [1–7]. DED has various symptoms that impair visual functioning and quality of life (QOL), thereby negatively affecting DED patients physically, socially, and psychologically [8–16].

Clinically, a diagnosis of DED requires objective findings from eye examination tests and subjective reports of dry eye symptoms. Many studies have found a discrepancy between subjective self-reported symptoms and objective findings in patients with DED [9,17]. For example, there has been no clear explanation for the presence of cases with measurable indications of dry eyes but no reported subjective symptoms; such cases lead to the improbable diagnosis that the person does not have DED.

Studies have also suggested that psychological factors are associated with DED symptoms [18–21]. Recently, depression has been found to be associated with DED symptoms in people with normal or mildly reduced tear production [18]. In addition, Li reported that the prevalence of anxiety and depression, as well as scores, were significantly higher in a DED group than in a control group in a case-control study [20]. With the recent rise in positive psychology research, the pursuit of happiness has become an important goal for many people [22] and the World Health Organization has increasingly emphasized happiness as an important component of health [23]. Well-being, also referred to as happiness, is a state of mind or feeling characterized by pleasure or satisfaction. Positive psychological factors such as happiness have been credited with improving human functioning and helping people live more successful lives [24].

Therefore, the association of happiness with DED should be also considered. However, until now, there has been little scientific exploration of the well-being of people with DED. It is hypothesized that subjective happiness may influence DED. The current study, exploring the relationship between the psychological factor of happiness and the objective and subjective findings of DED, was conducted at a company in Osaka, Japan.

## Methods

### Design, Setting, and Participants

We conducted a cross-sectional survey in 2011 among all the employees (N = 672; age range = 26–64 years old) of a company in Osaka, Japan. The sample consisted mainly of young and middle-aged Japanese office workers who used Visual Display Terminals (VDT). Written informed consent was obtained from all participants. Subjects were excluded from the study if they had undergone refractive surgery. The detailed study protocols were presented in a previous report [25]. The research protocol followed the tenets of the Declaration of Helsinki and was approved by the Institutional Review Board of the Ryogoku Eye Clinic, Tokyo, Japan.

### Outcome Measures

The outcome measures consisted of DED parameters (ocular surface epithelial damage, tear film breakup time (BUT), Schirmer test results, and a dry eye symptoms questionnaire) and the Subjective Happiness Scale (SHS).

#### Tear function tests and ocular surface evaluations

Ophthalmic examinations, including the assessment of conjunctival and corneal vital staining with lissamine green and fluorescein, tear film BUT, and the Schirmer test, were performed in order to determine the participants’ DED condition. Both eyes were examined and the scores of the eye with more serious DED (based on ratings of definitive-DED, probable-DED, or non-DED) were used in this study. When both eyes were the same grade, the eye that had a higher keratoconjunctival epithelial damage score was chosen; if the epithelial damage score was the same, then the eye with the shorter BUT was chosen. Finally, if both eyes were the same, the right eye was chosen. The detailed methods were given in our previous Osaka study [25]. All ophthalmic examinations were performed by ophthalmologists who specialized in DED.

The cornea and conjunctiva were assessed by fluorescein and lissamine green staining, using slit lamp. The eye was divided into three equal components of the nasal conjunctiva, cornea, and temporal conjunctiva; the maximum staining score for each area was three points. Overall, epithelial damage was scored on a scale of 0–9 points (Abnormal≥3) [26]. To determine the tear film BUT, fluorescein vital staining was performed; the participants were asked to blink three times to ensure adequate mixing of the fluorescein dye in the tears. The time interval between the last complete blink and the appearance of the first corneal dry spot was measured with a stopwatch, and the mean value of the three measurements was regarded as the tear film BUT in this study (Abnormal≤5 seconds).

The Schirmer test was performed without topical anesthesia, following all other examinations. Strips (Whatman No.41; Showa, Tokyo, Japan) were placed for 5 minutes at the outer one third of the temporal lower conjunctival fornix. The strips were then removed, and the length of filter paper that had been wetted (in mm) was recorded. To avoid the influence of conjunctivocorneal staining on the Schirmer test, we proceeded with that test after a 10-minute interval.(Abnormal value was considered as less than and equal to 5 mm).

#### Dry eye symptoms questionnaire

The widely used modified version of the Japanese dry eye symptoms questionnaire was completed by the participants. [12] The questionnaire included 12 questions pertaining to the diagnostic symptoms of DED. Response choices for each question about the symptoms were “constantly,” “often,” “sometimes,” and “never” **(** S1 Table). Each subjective symptom was given a score of 1 if the symptom rating was “constantly” or “often.” Participants were considered to possess symptoms of DED if any of the 12 symptoms received a rating of 1.

In addition to the 12-item dry eye symptoms questionnaire that was used to evaluate presence or absence of DED symptoms, a 29-item questionnaire was also used to evaluate the severity of symptoms associated with dry eyes (S2 Table). The questionnaire was a modification of the Ocular Surface Disease Index (OSDI). Each question was answered on a five-point Likert scale: 4 = “always”; 3 = “often”; 2 = “occasionally”; 1 = “rarely”; and 0 = “never.” The total symptom scores were calculated by computing the mean score of all answered items, which was transformed to a range of 0–100 (questionnaire score = [(sum of scores for all questions answered) × 100] / [total number of questions answered] × 4]) [27,28]. This scale has demonstrated adequate reliability and validity in previous studies [27,28]. The score from this questionnaire was considered a subjective measure of DED.

#### Diagnosis of DED

The diagnosis of DED was made according to the latest Japanese dry eye diagnostic criteria [26,27]: (1) presence of dry eye symptoms (according to the 12-item questionnaire), (2) presence of qualitative or quantitative disturbance of the tear film (the Schirmer test ≤5 mm or BUT ≤5 seconds), and (3) presence of conjunctivocorneal epithelial damage (total staining score ≥3 points). All three criteria were necessary for a “definite DED” diagnosis. Participants with two of the three criteria were given the diagnosis of “probable DED,” and a diagnosis of “non-DED” [26,27] was given if one or none of the three criteria were met.

The study further delineated the criteria from the above DED diagnosis according to an objective/subjective classification. Participants were assigned a status of (+) or (-) for objective assessments: Objective (+) indicates that the subject fulfilled (2) and/or (3), and Objective (-) indicates that the subject did not fulfill either (2) or (3). Participants were also assigned a status of (+) or (-) for subjective (symptoms) assessment: Subjective (+) indicates that the subject fulfilled (1), while Subjective (-) indicates that the subject did not fulfill (1). Subsequently, the participants were classified into four groups based on a combination of objective and subjective assessments: A = Objective (+) and Subjective (-); B = Objective (+) and Subjective (+); C = Objective (-) and Subjective (+); and D = Objective (-) and Subjective (-).

#### Subjective Happiness Scale

Subjective happiness was measured by the SHS, which was developed by Lyubomirsky and Lepper [29,30]. It is a four-item measure of subjective global happiness rated on a seven-point Likert scale **(** S3 Table **)** [31]. The current study used the Japanese version of SHS, which has established validity [31]. A single SHS score is the mean of the responses to the four items. SHS scores can range from 1 to 7, where a higher score indicates a higher level of happiness.

#### Other Measures

History or presence of common systemic diseases including hypertension, diabetes mellitus, depression, or other psychiatric disorders was determined by asking the subjects whether they had ever received a diagnosis of the disease by their physicians. Systemic medications, defined as medications prescribed by doctors (e.g., sleeping pills), were recorded.

### Statistical analysis

Statistical comparisons of the SHS score were made among the three DED diagnostic groups and the objective findings from dry eye examinations. Differences were analyzed by performing the analyses of variance (ANOVA) and Tukey’s comparisons. Associations between continuous variables were investigated with Pearson’s correlations. We used multiple linear regression analysis to adjust for possible confounders (gender, age, and BMI). The value of *p*<0.05 was set as the threshold of significance. All statistical analyses were performed using SAS software, version 9.2 (SAS Inc., Cary, NC).

## Results

The questionnaires and dry eye examinations were completed by 83.5% (561 of 672) of the participants. Characteristics of the participants and their SHS scores are summarized in Table 1. There were no participants with psychiatric disorders, including depression or anxiety, although 13 participants were taking prescribed sleep aids. Participants’ mean SHS score was 4.91 (*SD* = 1.01), and there was no significant difference between men and women. When the SHS scores were examined by age groupings (*F* = 6.11, *p*<0.05), those in their 30s had a lower SHS score than did the other age groups, except for those in their 40s (Table 1).

**Table 1:** Age, gender, and the Subjective Happiness Scale scores of the participants.

Gender	***n***	SHS score (Mean ± *SD*)	***p***-value
Male	374	4.91 ± 1.04	0.763
Female	187	4.88 ± 0.95	
**Age**			
26–29	34	5.3 ± 0.8	<0.001
30–39	155	4.7 ± 1.2	
40–49	231	4.8 ± 0.9	
50–59	122	5.1 ± 0.8	
60–64	19	5.4 ± 0.9	
Total	561	4.91 ± 1.01	

*Note*. p-values reported pertain to the results of an ANOVA; SD = standard deviation; SHS = Subjective Happiness Scale.

Dry eye symptoms scores were significantly lower in the “non-DED” group than the “definite DED” and “probable DED” groups (*F* = 57.34, *p*<0.001, Table 2). In contrast, no significant difference was observed in the SHS scores between the three diagnostic groups: “definite DED,” “probable DED,” and “non-DED.”

**Table 2:** Comparison of Subjective Happiness Scale and dry eye symptom scores.

	N	SHS score		Dry eye symptom score (29 items)	
		Mean ± *SD*	*p*-value	Mean ± *SD*	*p*-value
1. Definite DED	65	4.90 ± 1.04	0.917	31.8 ± 13.6	<0.001 1:2 *p* = 0.073, 1:3 *p* = 0.000, 2:3 *p* = 0.000
2. Probable DED	303	4.89 ± 1.00		28.1 ± 14.0	
3. Non-DED	193	4.92 ± 1.02		17.1 ± 9.1	

*Note*. DED = Dry eye disease; SHS = Subjective Happiness Scale

Next, we investigated the associations among the dry eye parameters and SHS. The percentage of the participants who had an abnormal conjunctivocorneal staining score was 16.0% (*n* = 90, mean = 1.09 and *SD* = 1.34). The percentage of those who had an abnormal BUT was 78.6% (*n* = 441, mean = 4.03 and *SD* = 2.48). The percentage of those who had abnormal Schirmer test results was 16.9% (*n* = 95, mean = 18.66 and *SD* = 11.73). The prevalence of positive dry eye symptoms was 71.1% (*n* = 399) according to the 12-item questionnaire, and the severity score, derived from the 29-item dry eye symptom questionnaire, was 24.7 (*SD* = 13.7).

SHS was significantly and negatively correlated with the dry eye symptoms score (*r* = -0.188, *p*<0.001) **(** Table 3). In contrast, none of the objective dry eye assessments was correlated with SHS scores **(** Table 3 **)**. After controlling for possible confounders (gender, age, BMI), there continued to be a significant association between the dry eye symptom score and the SHS score (*p*<0.001, Table 4)

**Table 3:** Correlation of SHS score with objective findings from dry eye examinations and subjective symptom scores.

Objective evaluations	Mean ± *SD*	*r*	*p*-value
Conjunctivocorneal staining score	1.09 ± 1.34	-0.007	0.866
BUT (seconds)	4.03 ± 2.48	-0.068	0.106
Schirmer test (mm)	18.66 ± 11.73	-0.006	0.886
**Subjective evaluation**			
Dry eye symptom score	24.7 ± 13.7	-0.188	<0.001

*Note*. SHS = Subjective Happiness Scale; SD = standard deviation; *r* = Pearson’s correlation coefficient; BUT = breakup time.

**Table 4:** Adjusted result of the Subjective Happiness Scale score: dry eye, gender, age, and body mass index.

	Subjective Happiness Scale score	
	Slope ± SE	*p*-value
Intercept	5.02 ± 0.38	<0.0001
Dry eye symptom score	-0.01 ± 0.00	<0.0001
Gender	0.09 ± 0.10	0.382
Age	0.09 ± 0.10	0.026
Body Mass Index	-0.01 ± 0.01	0.320

*Note*: SHS = Subjective Happiness Scale; SE = Standard error.

**Fig 1:**
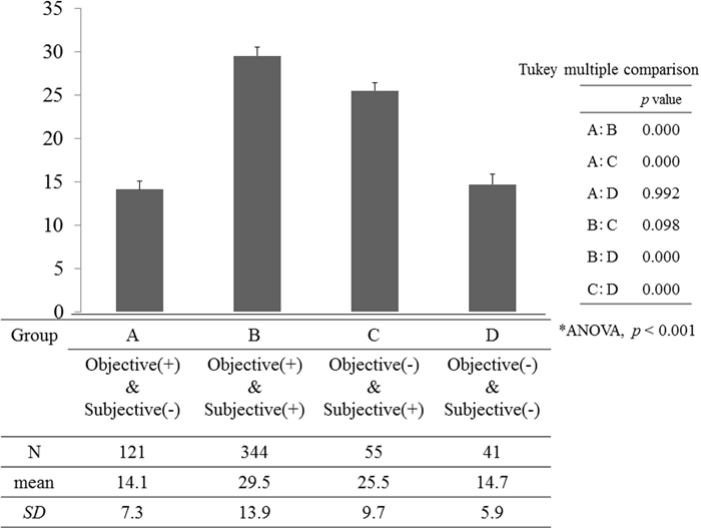
Comparison of dry eye symptom scores between objective and subjective classifications. SD = standard deviation.

In terms of the objective/subjective classification, the distribution of participants in the Objective (+)/Subjective (-), Objective (+)/Subjective (+), Objective (-)/Subjective (+), and Objective (-)/Subjective (-) groups was 21.6% (*n* = 121), 61.3% (*n* = 344), 9.8% (*n* = 55), and 7.3% (*n* = 41), respectively **(** Fig 1 **)**. Dry eye symptom scores were significantly different between the Objective/Subjective classification groups (*F* = 60.47, *p*<0.001). The dry eye symptom score of the Objective (+)/Subjective (+) group was significantly higher than were those of the Objective (+)/Subjective (-) and Objective (-)/Subjective (-) groups (*p*<0.001, Fig 1).

**Fig 2:**
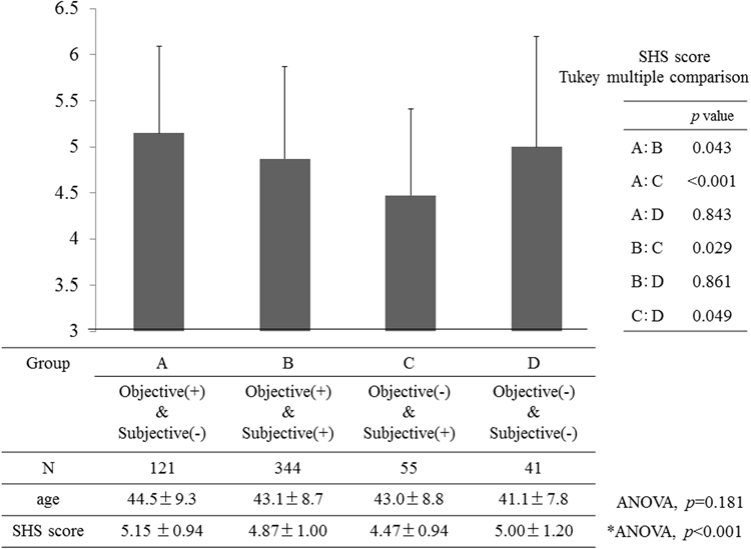
Comparison of SHS score between objective and subjective classifications. SD = standard deviation.

The SHS scores of the participants in the Objective (+)/Subjective (-) classification were significantly higher than were those of the Objective (-)/Subjective (+) and the Objective (+)/Subjective (+) groups (*F* = 6.18, *p*<0.05, Fig 2). In contrast, the SHS scores were significantly lower for the participants in the Objective (-)/Subjective (+) group than they were for participants in every other group, including the participants with “definite DED” (i.e., Objective (+)/Subjective (+)) (*p*<0.05, Fig 2).

## Discussion

In this study, we found that DED was associated with self-reported subjective happiness. Interestingly, a higher level of subjective happiness, as measured by the SHS, was significantly related to fewer self-reported dry eye symptoms, but the association was not significant between subjective happiness and all objective dry eye indicators. The result that participants with the lowest happiness scores reported symptoms of dry eyes but presented no objective indictors of DED deserves special attention.

In the study by Li and colleagues, anxiety scores were correlated with OSDI and education level in the DED group [20]. Neither anxiety nor depression scores were correlated with age, gender, the BUT, the Schirmer test, fluorescein staining score, or visual acuity [20]. Recently, Labbé et al. also reported that a depression score was correlated with dry eye symptoms, but not with the BUT, the Schirmer test, or corneal staining [32]. Furthermore, there have been numerous studies reporting the discrepancy between subjective and objective dry eye indicators. Since the ocular surface sensitivity is reduced in advanced ocular surface disease [33], and the tests currently used are far from being perfect, these are poorly associated with subjective symptoms [34–36]. To the best of our knowledge, our present study is the first to incorporate ‘subjective happiness’ as an additional aspect of consideration in objective and subjective determinants of DED severity.

**Fig 3:**
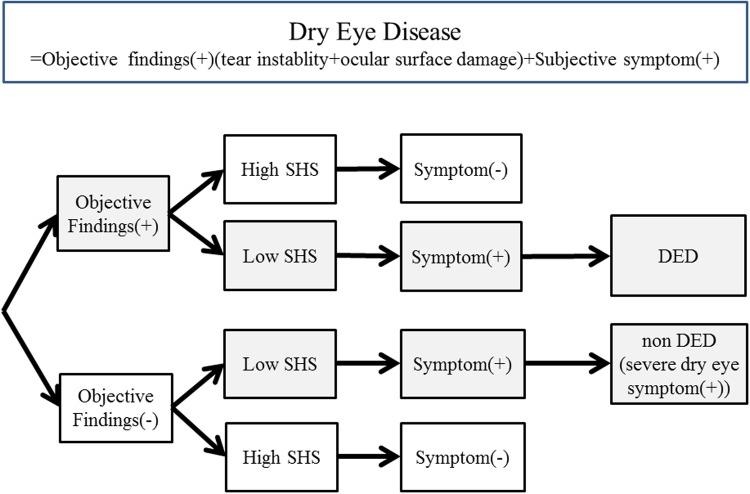
Hypothesis. SHS = Subjective Happiness Scale.

Subjective DED symptoms other than dryness of the eye, such as ocular fatigue and pain, may adversely affect quality of life (QOL) and quality of vision (QOV); thereby resulting in a lower SHS score. Another reason is that, there is a possibility that people with high SHS scores may tend not to focus on symptoms of dryness in their eyes and hence, are less likely to report symptoms of DED. In contrast, people with low SHS scores may be more conscious of and concerned about the symptoms of DED. In fact, more severe symptoms were reported in people with the lowest SHS scores; this occurred even when participants did not have positive results from objective eye examinations **(** Fig 3). To investigate DED that does not result in blindness, we deliberately selected a study group of young office workers using VDTs. It is of particular interest that we detected an association between SHS and subjective DED symptoms even in this population.

Positive psychology has been applied to depression, anxiety, and improvements in overall well-being. In other words, positive psychology strives "to find and nurture genius and talent" and "make normal life more fulfilling," rather than merely focusing on and treating mental illness [24]. Hence, when considering symptomatic conditions, the mental health status of participants is worth noting. It is already widely known DED reduce their QOL [8–16]. People who reported high levels of well-being seem to be able to sustain a high level of subjective happiness even during negative situations, and this might lead to a more positive evaluation of their life and a higher QOL. On a similar note, several studies have recently reported that the experience of pain, which is influenced by a complex mix of physical, psychological, social, and spiritual components, is also significantly correlated with happiness [37, 38]. There are many studies evaluating the relationship with subject symptom and happiness. Our study is the first study to present the association between dry eye symptoms and subjective happiness. We assume economic depression, interpersonal and marital relations, job stress, and job satisfaction may have an effect on the perception of happiness. In addition, dry eye symptoms may adversely affect subjective happiness. Therefore, an analytic observational study (such as a case-control) is necessary to address these confounding factors and evaluate the association between DED symptoms and SHS scores. Future research should also include an intervention study to provide confirmatory evidence for the association between DED symptoms and SHS and clarify causality.

While a causal relationship between SHS and DED symptoms could not be established from the results of this cross-sectional study, the association between DED symptoms and subjective happiness is apparent in these relatively young VDT workers (*p*<0.05). As previously stated, subjective dry eye symptoms are not solely restricted to feelings of ocular dryness, but comprise a plethora of bothersome and often debilitating symptoms such as ocular fatigue, foreign body sensation, blurred vision and pain. All of these dry eye symptoms can adversely affect QOL and QOV, and consequently, subjective happiness. Therapies aimed at relieving these subjective dry eye symptoms have the potential for improving SHS indices and overall patient happiness.

Future studies incorporating positive psychological intervention for DED are required to draw more definitive conclusions on the causality of the relationship between greater subjective happiness and lower symptoms in such symptomatic conditions. Also, we should do further investigation to dismiss all of the other possible options.

In conclusion, subjective happiness may have a stronger influence on DED symptoms than do ocular findings. We believe that the study has raised a novel viewpoint that supplements the standard approach of looking at DED from the pathogenesis perspective, as well as suggesting a possible treatment approach. Further research addressing the role of psychological factors in the presence and treatment of DED may prove promising.

## Supporting Information

S1 Table Dry eye symptoms questionnaire (12 items) . *Note*. 1 = Constantly; 2 = Often; 3 = Sometimes; 4 = Never.
(DOCX)

S2 Table Dry Eye Symptom (Severity) Questionnaire (29 items). *Note*. The answers to questions 1 to 29, except question 21, were rated on a 5-point scale: 4 = always; 3 = often; 2 = occasionally; 1 = rarely; 0 = never. The answers to question 21 were rating on a 5-point scale: 4 = not possible; 3 = with difficulty; 2 = moderately possible; 1 = probable; 0 = not a probable
(DOCX)

S3 Table Subjective Happiness Scale. A 4-item measure of global subjective happiness rated on a 7-point Likert scale (Lyubomirsky & Lepper, 1999). For each of the following statements and/or questions, please circle the point on the scale that you feel is most appropriate in describing you. *Note*. Item 4 is reverse coded.
(DOCX)
