# Supplementary material for: Associations between Subjective Happiness and Dry Eye Disease: A New Perspective from the Osaka Study
Source: PLoS One. 2015 Apr 1;10(4):e0123299. doi: 10.1371/journal.pone.0123299 (PMC4382322; doi:10.1371/journal.pone.0123299)
Supplement: S1 Table — . Note. 1 = Constantly; 2 = Often; 3 = Sometimes; 4 = Never. (DOCX) [file pone.0123299.s001.docx]

**S1 Table. Dry eye symptoms questionnaire (12 items)**

| Please check your symptoms |
| --- |
| 1. Ocular fatigue |
| 1. Discharge |
| 1. Foreign body sensation |
| 1. Heavy sensation |
| 1. Dry sensation |
| 1. Uncomfortable sensation |
| 1. Excess tearing |
| 1. Blurred vision |
| 1. Itching |
| 1. Sensitivity to the blight light |
| 1. Redness |
| 1. Pain |

*Note.* 1 = Constantly; 2 = Often; 3 = Sometimes; 4 = Never.
