# Supplementary material for: Associations between Subjective Happiness and Dry Eye Disease: A New Perspective from the Osaka Study
Source: PLoS One. 2015 Apr 1;10(4):e0123299. doi: 10.1371/journal.pone.0123299 (PMC4382322; doi:10.1371/journal.pone.0123299)
Supplement: S2 Table — Note. The answers to questions 1 to 29, except question 21, were rated on a 5-point scale: 4 = always; 3 = often; 2 = occasionally; 1 = rarely; 0 = never. The answers to question 21 were rating on a 5-point scale: 4 = not possible; 3 = with difficulty; 2 = moderately possible; 1 = probable; 0 = not a probable (DOCX) [file pone.0123299.s002.docx]

**S2 Table Dry Eye Symptom (Severity) Questionnaire (29 items)**

| Three-part questionnaire |
| --- |
| A. Symptom frequency severity scores for dryness |
| (1) Do you feel dryness in your eyes? |
| (2) Are your eyes uncomfortable? |
| (3) Do you experience foreign body sensation in your eyes? |
| (4) Do you have any pain in your eyes? |
| (5) Do you feel any eyestrain? |
| (6) Do you have any discomfort in your eyes? |
| (7) Are your eyes bloodshot? |
| (8) Do you have any itching in your eyes? |
| (9) Do you have any difﬁculty in opening your eyes? |
| (10) Do you have any irritation in your eyes? |
| (11) Do your eyes feel heavy? |
| (12) If you have any other symptoms, please write it in the parentheses beneath and circle the frequency |
| Total (1–12): ( )—A |
| B. Symptom frequency severity scores for visual disturbance |
| (13) Do you have hazy vision? |
| (14) Do you experience glare? |
| (15) While reading, do your dry eye symptoms become worse and does it become hard to continue reading? |
| (16) While driving, do your dry eye symptoms become worse and does it become difﬁcult to drive? |
| (17) While operating the computer, do your dry eye symptoms become worse and does it become difﬁcult to continue? |
| (18) While watching TV or movies, do your dry eye symptoms become worse and does it become difﬁcult to watch? |
| (19) Do you have any after-images? |
| (20) Do you think that blinking affects your sight? |
| (21) Can you keep your eyes open without blinking for 10 seconds or more? |
| Total (13–21): ( )—B |
| C. Symptom frequency severity scores for environmental and lifestyle factors: Do your dry eye symptoms become worse... |
| (22) When the wind is strong? |
| (23) When it is dry in winter or summer? |
| (24) When the air conditioning is on? |
| (25) While ﬂying? |
| (26) When you feel stressed in daily life? |
| (27) After alcohol consumption? |
| (28) When you smoke or are exposed to someone smoking next to you? |
| (29) When you are wearing contact lenses? |
| Total (22–29): ( )—C |

*Note.*

The answers to questions 1 to 29, except question 21, were rated on a 5-point scale: 4 = always; 3 = often; 2 = occasionally; 1 = rarely; 0 = never.

The answers to question 21 were rating on a 5-point scale: 4 = not possible; 3 = with difﬁculty; 2 = moderately possible; 1 = probable; 0 = not a probable
