# Supplementary material for: Associations between Subjective Happiness and Dry Eye Disease: A New Perspective from the Osaka Study
Source: PLoS One. 2015 Apr 1;10(4):e0123299. doi: 10.1371/journal.pone.0123299 (PMC4382322; doi:10.1371/journal.pone.0123299)
Supplement: S3 Table — A 4-item measure of global subjective happiness rated on a 7-point Likert scale (Lyubomirsky & Lepper, 1999). For each of the following statements and/or questions, please circle the point on the scale that you feel is most appropriate in describing you. Note. Item 4 is reverse coded. (DOCX) [file pone.0123299.s003.docx]

**S3 Table. Subjective Happiness Scale**

A 4-item measure of global subjective happiness rated on a 7-point Likert scale (Lyubomirsky & Lepper, 1999)

For each of the following statements and/or questions, please circle the point on the scale that you feel is most appropriate in describing you.

| 1. In general, I consider myself not a very happy person or a very happy person. |
| --- |
| Not a very happy 1,2, 3,4,5,6,7 a very happy person |
| 1. Compared to most of my peers, I consider myself less happy or happier. |
| Less happy 1,2, 3, 4,5,6,7 more happy |
| 1. Some people are generally very happy. They enjoy life regardless of what is going on, getting the most out of everything. To what extent does this characterization describe you? |
| Not at all 1,2, 3, 4,5,6,7 a great deal |
| 1. Some people are generally not very happy. Although they are not depressed, they never seem as happy as they might be. To what extent does this characterization describe you? |
| Not at all 1,2, 3, 4,5,6,7 a great deal |

*Note.* Item 4 is reverse coded.
